# Organizational Support Experiences of Care Home and Home Care Staff in Sweden, Italy, Germany and the United Kingdom during the COVID-19 Pandemic

**DOI:** 10.3390/healthcare9060767

**Published:** 2021-06-19

**Authors:** Connie Lethin, Andrea Kenkmann, Carlos Chiatti, Jonas Christensen, Tamara Backhouse, Anne Killett, Oliver Fisher, Agneta Malmgren Fänge

**Affiliations:** 1 Department of Health Sciences, Faculty of Medicine, Lund University, 221 00 Lund, Sweden; connie.lethin@med.lu.se; 2 Clinical Memory Research Unit, Department of Clinical Sciences, Faculty of Medicine, Lund University, 221 00 Lund, Sweden; 3 Center for Aging, Katholische Stiftungshochschule, 81667 München, Germany; Andrea.kenkmann@ksh-m.de; 4 Tech4Care srl, 60015 Falconara Marittima, Italy; c.chiatti@tech4care.it; 5 Department of Social work, Faculty of Health and Society, Malmö University, 21119 Malmö, Sweden; jonas.christensen@mau.se; 6 School of Health Sciences, University of East Anglia, Norwich NR4 7TJ, UK; tamara.backhouse@uea.ac.uk (T.B.); a.killett@uea.ac.uk (A.K.); 7 Centre for Socio-Economic Research on Ageing, IRCCS INRCA-National Institute of Health and Science on Ageing, 60124 Ancona, Italy; o.fisher@inrca.it; 8 Department of Economics and Social Sciences, Università Politecnica delle Marche, 60121 Ancona, Italy

**Keywords:** COVID-19, care home, home care, organizational support, staff experience, survey

## Abstract

The COVID-19 pandemic has affected care workers all over the globe, as older and more vulnerable people face a high risk of developing severe symptoms and dying from the virus infection. The aim of this study was to compare staff experiences of stress and anxiety as well as internal and external organizational support in Sweden, Italy, Germany, and the United Kingdom (UK) in order to determine how care staff were affected by the pandemic. A 29-item online questionnaire was used to collect data from care staff respondents: management (*n* = 136), nurses (*n* = 132), nursing assistants (*n* = 195), and other healthcare staff working in these organizations (*n* = 132). Stress and anxiety levels were highest in the UK and Germany, with Swedish staff showing the least stress. Internal and external support only partially explain the outcomes. Striking discrepancies between different staff groups’ assessment of organizational support as well as a lack of staff voice in the UK and Germany could be key factors in understanding staff’s stress levels during the pandemic. Structural, political, cultural, and economic factors play a significant role, not only factors within the care organization or in the immediate context.

## 1. Introduction

The COVID-19 [1] pandemic has affected care staff worldwide. It is estimated that, on average, half of all COVID-19-related deaths during the first wave of the pandemic occurred in care homes [2,3]. Alacevich et al. 2020 suggest that care homes were also one catalyst in spreading the disease in their local communities [4].

While many countries focused their initial support on hospitals, care homes and home care did not receive the same attention. Personal protective equipment (PPE) and testing facilities were often missing or delayed [3,5], and when testing became available, testing strategies were not optimized [6]. In addition, staff had to bear the burden of additional infection control measures, staff shortages, worries about their own and residents’ health, and excess deaths [2,7].

Shreffer et al. [8] found, in their scoping review on the impact of the pandemic on health workers, consistent evidence for increased stress and anxiety levels among staff. Although the included studies relate to health workers in the acute sector, White et al. [9] identified emotional burden and burnout in nursing home staff in the United States. It is likely that care home and home care staff in Europe have been similarly affected.

Stress has been a priority in the European Union (EU) for several years in relation to work life conditions. Research has also shown that stress is correlated to work conditions overall in the care sector [10,11]. Unsupportive management is seen as a source of occupational stress for nursing staff [12], and poor management can contribute to inadequate care [13]. Rajamohan et al. [14] found that job satisfaction is highest amongst nursing staff when they are able to deliver consistent high-quality care to residents and thus improve their quality of life. Supportive management, but also training opportunities play a key factor in enhancing job satisfaction and increase the likelihood of staff retention [14,15,16,17]. It is therefore important to ask about the support and training that care home and home care staff received during the COVID-19 pandemic, as such training possibly contributes to the health, well-being, and job satisfaction of care staff and, consequently, the quality of life of the people cared for.

**Figure 1:**
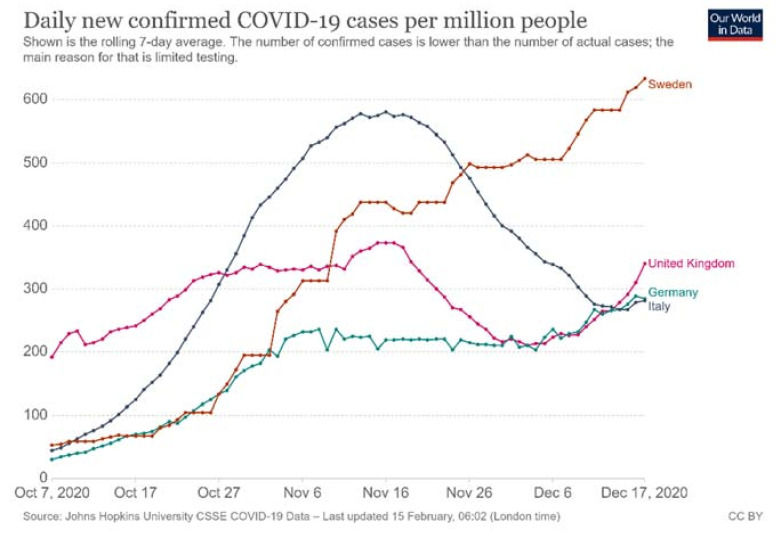
Daily confirmed COVID-19 cases during 7 October–7 December 2020.

**Figure 2:**
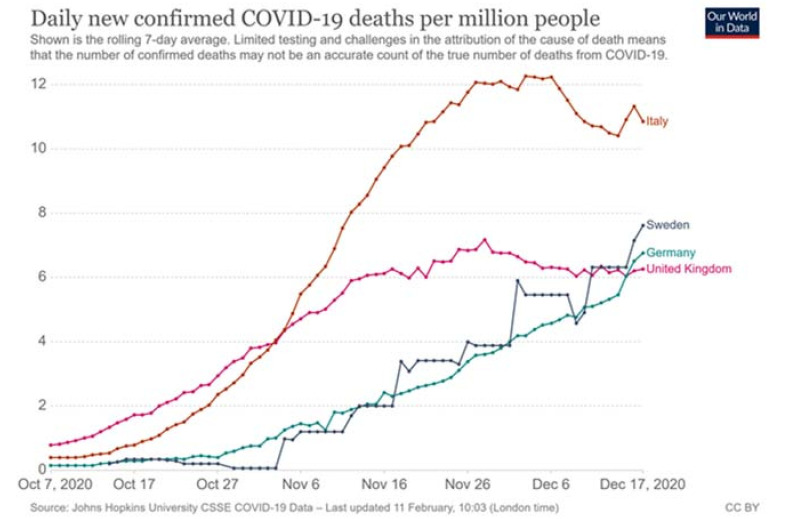
Daily confirmed COVID-19-related deaths during 7 October–7 December 2020.

Within the EU, different countries were differently affected by the pandemic. Sweden, Italy, Germany, and the United Kingdom (UK) were all heavily affected; in total, they had 5,945,070 confirmed COVID-19 cases and 172,035 related deaths by 21 December 2020 (John Hopkins University, 21 December 2020). As we can see in Figure 1 and Figure 2, confirmed COVID-19 cases and COVID-19-related deaths do not correlate to each other in similar ways in these four countries, indicating that the pandemic has been managed differently at national level.

Across Europe, the effect of an aging population varies due to differing socio-economic development and welfare logics, such as in the Swedish, UK, Italian, and German welfare systems [18]. In Sweden, all citizens have equal access to all welfare benefits financed through a graduated tax system based on collective solidarity. The national variations are relatively great due to the relative autonomous position of the local authorities (municipalities), which are responsible for the provision and organization of the home care and care home services for people aged 65 years or older. In the UK, entirely publicly funded health service and the organizing of care for older people is relatively more centralized than in Sweden, leaving local authorities with fewer options to design the care according to local conditions, described as an integrated health and social care model [19]. The majority of care services are privately run, and the government sponsors the Care Quality Commission [20], an independent public body, to regulate all health and social care services with local authorities monitoring quality where they are funding care. The Italian welfare state is based upon the corporatist-conservative model. Social services to older people, people with disabilities, and needy families are dealt with by local authorities, sometimes with the assistance of volunteer associations and no-profit social service cooperatives [21]. The Italian National Healthcare System is tax-funded, both by public and private providers, and mainly managed by 21 Regions and the autonomous provinces. Each region then has its Local Health Agencies (ASLs), which are in charge of providing care in hospitals, at home and in residential structures for older people [22].

In Germany, the system to fund Long Term Care, introduced in 1995, is based on a social partnership welfare state model linked to the principle of subsidiarity and the dominance of social insurance schemes where care is closely embedded in the social insurance system. Long Term Care insurance is mandatory for citizens, and although traditionally non-statutory welfare services were key in providing services, increasingly private companies are occupying a large share of the German care market. The case is further complicated by insurance companies monitoring care homes as well as the regional authorities.

Accordingly, although countries issued national guidelines during the pandemic, the support structures for care homes and home care organizations can lie at a regional or local level, and care homes and home care are provided by a spectrum of different care organizations.

The combination of the differences in the management of the pandemic and the way care is organized in these countries, along with how they affect each other, leads to a complex picture of the diversity of how care is organized and structured between the countries as well as within. Moreover, the decision-making processes might vary on management and staff levels which also adds to this complexity.

The overarching aim of the current study was to start unravelling some of these complexities by analyzing how staff working in care homes and home care services have experienced support within these organizations as well as from their local authorities, communities, and individuals. Another aim was to investigate the level of stress and anxiety among the care staff as well as to identify which factors were associated with these levels. Our first hypothesis was that the pandemic has affected care staff differently in each country, and the second hypothesis was that staff experience lower levels of stress and anxiety if they receive good support from within their organizations and if the organizations receive external support.

## 2. Materials and Methods

### 2.1. Study Design

This study applied an exploratory, cross-sectional design using an online survey in four European countries, Germany, Italy, Sweden, and the UK.

### 2.2. Data Collection

#### 2.2.1. Questionnaire

A questionnaire containing 29 items was designed. Information about study purpose, data management, analysis, and dissemination as well as contact details of the responsible researchers were provided, and by filling in the questionnaire the respondents simultaneously gave their informed consent to participation. The questionnaire was anonymous. The first part of the questions comprised demographic and personal data (six items related to age, gender, role in the organization, type and location of organization the participants worked in). In the second part of the questions, first a single question on the impact of the pandemic on stress and anxiety level was asked, measured on a five-point scale ranging from 1 = no impact to 5 = very strong impact. Thereafter, the participants were asked to indicate their level of agreement to 22 questions using a five-point scale from 1 = strongly agree to 5 = strongly disagree. Statements were organized in four sections: “Support from within the organization” and “External support for the organization”, “Learning experiences”, and “Outlook for the future”. Data from the first two sections are reported in this paper. The questions were developed specifically for this study, based on expert opinions in the research team as well as gaps in the knowledge about care home staffs’ work situation during the pandemic.

The questionnaire was designed in English and subsequently translated into Italian, German, and Swedish based on the English version. Particular care was taken to keep the same variables in all questionnaires whilst ensuring that the phrasing worked in the national context. In the questionnaires of Italy, Germany, and the UK, a regional variable was inserted, and in the English and German questionnaires there was furthermore a distinction between home care and care home staff. Before data collection, the translated versions were verified by each national team, internally and externally, until consent was reached.

Study data were collected and managed using REDCap tools hosted at Lund University, Faculty of Medicine, Lund, Sweden. REDCap is a secure, web-based software platform designed to support data capture for research studies, providing (1) an intuitive interface for validated data capture; (2) audit trails for tracking data manipulation and export procedures; (3) automated export procedures for seamless data downloads to common statistical packages; and (4) procedures for data integration and interoperability with external sources [23,24].

#### 2.2.2. Procedure

Convenience and snowball sampling procedures were applied to access this hard-to-engage group. Authors disseminated a link to the survey through social media (Twitter, LinkedIn, and Facebook, including relevant care staff groups on Facebook) and distributed through the authors’ professional networks and other relevant media such as newsletters for care workers. Study information was set out at the beginning of the survey questionnaire and consent from respondents was provided via completion of the survey. Data were collected during 7 October–17 December 2020. All responses were anonymous.

### 2.3. Data Analysis

Before the data analysis, data collected using the five-graded scale were transformed so that 1 = strongly disagree and 5 = strongly agree. Data were analyzed item-wise for each country and profession, separately and in total and for differences between the countries. Differences between countries were calculated using ANOVA tests. Regression analyses were performed to investigate independent factors associated with stress and anxiety. Potential factors were tested in the model if they had a *p*-value ≤ 0.25 following the results of the bivariate analysis and inserted in the model using a step-forward approach. Multicollinearity was controlled for by testing each independent variable against one another via linear regression analysis. The data were analyzed in SPSS 27.0 and STATA 14. *p*-values ≤ 0.05 were considered significant.

In the final analytical part of the paper, to summarize factors affecting stress levels average total scores for internal and external support were calculated for the four countries and all values <3.30 were defined as low, 3.69–3.30 as medium, 4.00–3.70 as high, and values >4.00 were defined as very high. As an indication of the different experiences of the pandemic in the four countries, the cumulative COVID-19-related deaths per million population by the beginning of the study was added to factors. Values <200 were defined as low, 399–200 as medium, 600–400 as high, and values >600 were defined as very high.

## 3. Results

The study sample contained considerable differences across the four countries as Table 1 shows. Respondents in all countries were predominantly female, while the respondents from Germany were younger than in the other countries. In Sweden and Italy, over half of the respondents were nursing assistants, but in Germany registered nurses were in the majority. The UK sample has an overrepresentation of other professions. In the Swedish sample, the vast majority worked in the public sector, whereas in the UK and Italy the majority were working in the private sector. The German sample was spread more evenly over type of organization.

### 3.1. Stress and Anxiety Levels

As Table 2 shows, the staff in all countries experienced significantly different levels of stress and anxiety, except amongst nursing assistants. Total stress and anxiety levels were highest in the UK followed by Germany and Italy, with Sweden reporting the lowest. In Germany and the UK, managers/coordinators perceived the pandemic as significantly more stressful than their Swedish and Italian counterparts. Registered nurses in the UK felt the most stressed of all professions in all countries, whereas their Swedish colleagues were the least stressed. In Sweden, all staff had similar perceptions of stress and anxiety levels, but the spread of replies within the registered nurse group was greater than in the UK and Germany. In Germany, there was a striking gap between stress levels of managers/coordinators and the other professions, and the Italian registered nurses experienced a higher level of stress than other Italian staff groups.

### 3.2. Internal Support for the Staff from within the Organization

Table 3 shows that levels of perceived management support varied across staff groups as well as countries, but also within groups. Across all results, the Swedish staff reported the most positive perception of internal support. Whereas the managers/coordinators in all four countries agreed that available support was good, registered nurses professed less support with Italian and German nurses reporting the least. Although the response patterns across staff groups and countries was similar for both items, overall staff in all countries indicated a higher level of agreement to having received clear guidelines compared to support from the managers/coordinators. Whereas in Italy and Sweden, mean agreement values on their voice being heard were similar to those regarding management support for all staff groups, in Germany and the UK we observed a drop in agreement for registered nurses, nursing assistants, and other staff, suggesting that these groups’ expertise was not valued. In Sweden and Italy there was agreement from all staff groups that adequate PPE had been available, but in Germany and the UK opinions varied considerably. In Sweden and Germany there was agreement across all staff groups that holiday entitlement could have been taken during the pandemic, while in the UK and in Italy the opinions were more scattered.

### 3.3. External Support for the Organization

As shown in Table 4, Swedish managers/coordinators, nursing assistants, and other staff reported the highest agreement that their organizations received clear guidelines from external regulators/government, whereas registered nurses agreed slightly less with this statement. In the UK, staff also indicated that external guidelines were clear, but managers/coordinators had a slightly lower agreement than the other staff groups. In Italy and Germany, managers/coordinators did not agree that organizations received clear guidelines; registered nurses shared these concerns to some extent, but nursing assistants in these countries thought that guidelines had been clear. Staff in both Sweden and the UK indicated that community support had been good, but their German colleagues agreed to a lesser extent. Whereas staff in Sweden and the UK felt positive about the received community support, those in Germany and Italy agreed less.

### 3.4. Factors Associated with Stress and Anxiety Levels

As shown in Table 5, the total sample stress and anxiety levels were significantly associated with being a care staff in the UK and Germany, in particular being a registered nurse or a manager/coordinator. In the total sample, having support from the management was associated with lower stress and anxiety levels. Age was not significantly associated with stress levels for managers/coordinators and registered nurses, but older nursing assistants and other care staff were the least stressed. No association with gender was found. Table 6 shows a summary of the data and includes the impact of the pandemic on stress and anxiety in the four countries at the beginning of the data collection phase. The results show inconsistencies between countries; these would remain if we were to break down the summary to staff group levels.

## 4. Discussion

This study explored the experiences and perceptions of care staff in Sweden, Italy, Germany, and the UK around the time of the COVID-19 second wave in each country. The results confirm our first hypothesis that the pandemic has affected care staff differently across countries but also across staff categories within each country. Our second hypothesis was that staff would experience lower levels of stress and anxiety if they received good support from within their organizations and if the organizations received external support. The results fit the second hypothesis in relation to Sweden, Germany, and Italy; however, the data from the UK disconfirm the hypothesis. The results suggest that the experience of the pandemic could be only one factor amongst others to understand staff stress and anxiety levels.

In this study, we found significant differences in perceived internal support across countries but also across staff groups, with Swedish staff having the most positive perception of support from the management and having their voices heard. One potential explanation could be the way leadership was enacted in the care organizations in the different countries. It has been pointed out that when a situation is uncertain and there is a low level of agreement among experts and politicians and among countries on what to do and what will come next, as with the COVID-19 pandemic, there is little use for a leader to focus on details and control. Instead, a decisive, visible, and communicative leadership targeting the goals and strategies [25], focusing on communication, collaboration, coordination, and providing support [26] is crucial. In order to facilitate communication and support, the majority of the Swedish municipalities re-organized their care teams early on, with those working with coronavirus-infected clients only in separate teams. Whereas in Sweden (and Italy), mean agreement values on their voice being heard were higher in all staff groups, compared to Germany and the UK our results indicate that this strategy might explain some of the differences. Having access to adequate PPE generates a sense of support and care for each staff member. The fact that in Sweden and Italy there was agreement of all staff groups that adequate PPE had been available, but in Germany and the UK opinions varied considerably, could be related to a general feeling of being listened to. This may also be related to the agreement across all staff groups that holiday entitlements could have been taken during the pandemic, even if the picture was a little bit scattered.

When it comes to the experience of external support, significant differences between countries were found, with Swedish staff again reporting the most support in the form of clear guidelines but also by support from the community. This finding could be linked to the previously discussed perception of having support from within the organization, since guidelines for care are most commonly communicated via managers. Registered nurses in Italy and the UK reported higher stress than other staff groups in their countries. Conversely, registered nurses in Sweden reported the lowest stress among all staff groups. Whereas Ley et al. [27] found in their study on nursing staff in the acute sector that organizational support had a direct impact on stress levels, Islam et al. [28] found that the type of care home (nursing or residential) could affect stress as they noted that registered nurses received more psychological demands [28]. Dunn et al. identified several organizational factors contributing to stress among professionals in care homes. Among these were “shortage of essential resources, not enough people per shift, feeling undervalued by management” [29], (p.177). However, our data suggest a more complex picture as shortage of essential resources is not a contributing factor for stress in our dataset, as in all countries adequate PPE was reported to be provided. The lack of staff appreciation, however, can be an important source of stress.

Our results imply that care staff stress levels have been high during the pandemic. This is consistent with other studies internationally, which identified complex stresses [9] and COVID-19-related shortages of professionals in the organization [30,31]. Professionals in the UK indicated high stress levels even though organizational support was perceived as good. For Sweden, one possible explanation might be the professional mandate nurses and other registered healthcare professionals in Sweden have, allowing them to take qualified patient care decisions independently. On one side, it increases the control over one’s work situation, but on the other side decision-making can be very stressful, in particular with time constraints and when experience is limited, such as with the SARS-CoV-2 virus. It could also be that in Sweden, employment rights are more far reaching than in the other countries included in our study. Most of all, it should be kept in mind that their stress levels were still high.

The experience and perception of stress and anxiety among our participants cannot, of course, be explained by the COVID-19 pandemic only. For example, the stress and anxiety among the UK staff differed from their colleagues in the other countries, which could be due to factors such as changes in staffing due to the UK nearing the time of Brexit during the time of our survey [32,33]. Alternatively, factors relating to care staff stress are more complex than just being a result of pandemic-related experiences, particularly since staff in the UK and Germany show the highest level of stress and anxiety and their experience of the COVID-19 pandemic up to the point of data collection could not have been more different.

Structural, policy, and political level decisions play an important role in how different countries have managed the pandemic and its impact on the society at large and the health and social care services. In addition, the role of media has been unprecedented during the pandemic, with misleading information significantly leading to distrust. Leading by example is considered crucial in times of crisis; however, leaders in the UK have been pointed out as having failed to do so [34], and in the other countries involved in this study, similar discussions have been frequent. Experiences of stress and support factors may, to a large extent, be affected through political decision-making processes and traditions, digital and economical aspects, as well as social and cultural traditions. As highlighted by the WHO [35], an integrated whole-of-society approach is crucial for setting priorities to maintain and improve health among Europe’s citizens, and Vinkers et al. [36] point out poverty, risk of unemployment, and poor access to healthcare and care homes as factors negatively affecting stress and anxiety. Some jobs in these sectors, for example nursing assistants, are low paid and the employment conditions are insecure, which increases the risk of spreading infections to clients and patients, as was seen in the UK [37]. Other reasons put forward include a highly fragmented healthcare and care services systems with very little sharing of information between the different actors [38]. With that said, experiences such as those reported in this study are likely to have been affected by factors and events in the society at large.

### Limitations

There are of course limitations in our study, and the results should be interpreted with caution. As the questionnaire for this hard-to-engage group was kept deliberately short, some relevant information, such as COVID-19 outbreaks in their organizations, staff shortages, and the size of the care home, was not collected. These aspects could potentially be key factors for stress levels and information about them would have supported the interpretation of our findings and added to the knowledge about the care professionals’ working conditions [30].

Additionally, the study does not provide enough context to assess whether hierarchical structures, communication cultures, or decision-making processes caused the discrepancies in reported experiences. As argued by Killett et al. [39], organizational cultures in care homes are complex and contextual, and the data suggest that there might be different organizational cultures in the four participating countries. Such information was, however, not included in our study.

The time of the data collection (10 July–17 December 2020) coincided with the beginning of the second wave of the pandemic in Italy, Sweden, and Germany. In the UK, the second wave had started a month earlier, which might have contributed to differences across countries to some extent. Sample sizes and the composition of samples in terms of professional group and organization varied across countries, with some subgroups being very small. The sample sizes in Germany and Italy were lower than in Sweden and the UK, which of course is a limitation here. Comparison data showing the staff experiences in these countries before COVID-19 would have been useful.

The UK sample has an overrepresentation of ‘other professionals’ (44.6%), which is likely to be due to care assistants not identifying with the term nursing assistant. Some differences can be explained by different staff requirements and different roles of staff in their organizations (e.g., in Sweden with the expectation of registered nurses to work more independently than in Germany, and in the UK being a registered nurse is a requirement only if a care home is registered to provide ‘care home services with nursing’ [40]) but the sampling strategy is likely to have affected the sample as well.

The questionnaire was developed in English. Translations into German, Italian, and Swedish. They were intended to be as accurate as possible, but competences and aspects associated with words that are included in different professional areas of competence (for example, nurse), or in terms used (care home, nursing home, etc.) can differ between countries. The questionnaire should be able to be used in all countries despite their differences in organization, which means that some detailed information also had to be left out.

## 5. Conclusions

Care staff experienced the COVID-19 pandemic differently in Sweden, Italy, Germany, and the UK. A mixed and complex picture of experiences of stress, anxiety, and internal as well as external support emerged. Discrepancies between different staff groups’ assessment of organizational support as well as a lack of voices being heard in some countries but not in others could be key factors in understanding staff’s stress and anxiety levels during the pandemic. We also believe that economic, cultural, and political factors, and potentially media, play a significant role on the care staff’s experiences during the pandemic, and not only factors related to their direct work situation and the decisions they can make there.

Future research should focus on examining care staff’s learning process and possibilities as well as the obtainment of new skills throughout the pandemic. Research should also focus on their perceptions about whether care practices or the status of care staff will change in the future.

## Figures and Tables

**Table 1:** Description of sample.

		Sweden *n* = 212	Italy *n* = 103	Germany *n* = 120	UK *n* = 167	Total *n* = 602
Gender, *n* (%)	Male	18 (8.8)	18 (17.5)	22 (18.3)	6 (3.6)	64 (10.8)
	Female	186 (91.2)	85 (82.5)	97 (80.8)	161 (96.4)	529 (89.0)
	Non-binary/third gender	0 (0.0)	0 (0.0)	1 (0.8)	0 (0.0)	1 (0.2)
Age, Mean (SD)		45.74 (12.1)	44.77 (11.3)	38.69 (12.9)	44.80 (12.0)	43.86 (12.4)
Professional role, *n* (%)	Managers/coordinator	16 (7.7)	24 (23.5)	31 (26.1)	65 (39.2)	136 (22.8)
	Registered nurse	36 (17.3)	12 (11.8)	70 (58.8)	14 (8.4)	132 (22.2)
	Nursing assistant	119 (57.2)	52 (51.0)	11 (9.2)	13 (7.8)	195 (32.8)
	Other care staff	37 (17.8)	14 (13.7)	7 (5.9)	74 (44.6)	132 (22.2)
Type of organization	Public	190 (93.1)	33 (32.0)	44 (37.0)	13 (7.8)	280 (47.2)
	Private	13 (6.4)	67 (65.0)	41 (34.4)	151 (90.4)	272 (45.9)
	Non-profit/voluntary	1 (0.5)	3 (3.0)	34 (28.6)	3 (1.8)	41 (6.9)

*n* = number; SD = standard deviation.

**Table 2:** Stress and anxiety levels by profession and country.

	Sweden		Italy		Germany		UK		Total		*p* Value
	*n*	M (SD)	*n*	M (SD)	*n*	M (SD)	*n*	M (SD)	*n*	M (SD)	
Manager/ coordinator	16	2.94 (1.06)	24	3.58 (1.02)	31	4.23 (0.81)	65	4.02 (0.93)	136	3.86 (1.01)	**0.001**
Registered nurse	36	2.81 (1.31)	12	3.83 (1.03)	70	3.6 (1.00)	14	4.29 (0.73)	132	3.48 (1.16)	**0.001**
Nursing assistant	117	3.26 (1.06)	51	3.24 (1.14)	11	3.55 (0.82)	13	3.92 (0.86)	192	3.32 (1.07)	0.153
Other care staff	36	3.11 (1.04)	14	3.43 (0.76)	7	3.29 (1.11)	71	3.92 (1.05)	128	3.6 (1.08)	**0.002**
Total	206	3.14 (1.12)	101	3.42 (1.06)	119	3.74 (0.98)	164	3.99 (0.96)	590	3.54 (1.09)	**<0.001**

*n *= number; M = mean; SD = standard deviation. Significant values in bold (*p* ≤ 0.05).

**Table 3:** Internal support from organization by profession and country.

	Sweden		Italy		Germany		UK		Total		*p* Value
	*n*	M (SD)	*n*	M (SD)	*n*	M (SD)	*n*	M (SD)	*n*	M (SD)	
I received clear guidelines from my organization											
Manager/coordinator	16	4.25 (0.68)	23	3.43 (1.20)	31	3.58 (1.43)	64	4.22 (1.13)	134	3.94 (1.22)	**0.010**
Registered nurse	36	3.64 (1.07)	12	3.42 (1.08)	70	3.44 (1.00)	14	4.14 (0.77)	132	3.57 (1.02)	0.114
Nursing assistant	119	4.29 (0.99)	51	4.33 (0.97)	11	3.36 (0.81)	13	3.46 (1.27)	194	4.2 (1.03)	**0.001**
Other care staff	37	4.3 (0.78)	14	3.57 (1.22)	7	3.71 (1.7)	74	4.15 (1.22)	132	4.11 (1.15)	0.177
Total	210	4.17 (0.98)	101	3.89 (1.16)	119	3.49 (1.15)	166	4.13 (1.17)	596	3.97 (1.13)	**<0.001**
I received adequate support from management											
Manager/coordinator	16	4 (1.10)	22	4 (1.11)	28	4 (1.12)	63	4.24 (1.25)	129	4.12 (1.18)	0.729
Registered nurse	36	3.58 (1.13)	12	3.25 (1.29)	69	3.26 (1.42)	14	3.93 (1.21)	131	3.42 (1.32)	0.281
Nursing assistant	116	3.85 (1.11)	47	3.98 (1.26)	11	2.73 (1.68)	13	3.15 (1.41)	187	3.77 (1.24)	**0.005**
Other care staff	37	3.97 (1.14)	14	3.29 (1.14)	7	2.57 (1.27)	72	3.78 (1.47)	130	3.72 (1.37)	**0.049**
Total	207	3.85 (1.12)	96	3.77 (1.24)	115	3.35 (1.42)	163	3.93 (1.38)	581	3.76 (1.29)	**0.001**
I was provided with adequate personal protective equipment											
Manager/coordinator	16	4.19 (1.22)	23	3.7 (1.40)	31	4.13 (0.99)	64	4.36 (1.15)	134	4.17 (1.18)	0.144
Registered nurse	36	4.11 (0.98)	12	3.58 (1.31)	70	3.04 (1.29)	14	3.79 (1.12)	132	3.46 (1.27)	**0.000**
Nursing assistant	118	4 (1.08)	48	4.17 (1.14)	11	3.36 (1.5)	13	3.46 (1.39)	190	3.97 (1.15)	0.070
Other care staff	37	4.24 (0.93)	14	3.64 (1.34)	7	3.57 (1.27)	73	3.96 (1.27)	131	3.98 (1.20)	0.070
Total	209	4.09 (1.04)	98	3.92 (1.26)	119	3.39 (1.31)	165	4.07 (1.24)	591	3.91 (1.22)	**<0.001**
I received adequate training											
Manager/coordinator	16	4 (1.03)	22	3.73 (1.08)	31	3.94 (1.36)	64	4.34 (1.06)	133	4.11 (1.15)	0.113
Registered nurse	35	3.77 (1.06)	12	3.42 (1.51)	69	2.96 (1.19)	14	3.57 (1.02)	130	3.28 (1.22)	**0.008**
Nursing assistant	117	3.77 (1.09)	50	4.06 (1.36)	11	3.36 (1.36)	13	3 (1.41)	191	3.77 (1.22)	**0.026**
Other care staff	36	4.03 (0.97)	14	3.5 (1.22)	7	2.71 (1.50)	72	3.74 (1.42)	129	3.74 (1.31)	0.090
Total	206	3.84 (1.06)	99	3.81 (1.31)	118	3.24 (1.33)	164	3.91 (1.31)	587	3.73 (1.25)	**<0.001**
My voice was listened to											
Manager/coordinator	16	3.94 (1.06)	22	4.14 (1.08)	31	4.26 (1.09)	63	4.24 (1.13)	132	4.19 (1.10)	0.772
Registered nurse	34	3.59 (1.02)	12	3.75 (0.87)	67	2.85 (1.29)	14	3.36 (1.08)	127	3.19 (1.21)	**0.007**
Nursing assistant	113	3.71 (1.15)	46	3.8 (1.28)	10	2.3 (1.06)	13	2.54 (0.97)	182	3.57 (1.24)	**<0.001**
Other care staff	36	3.83 (1.21)	12	3.17 (1.47)	6	2.5 (1.22)	72	3.32 (1.47)	126	3.41 (1.41)	0.092
Total	201	3.74 (1.13)	93	3.8 (1.22)	114	3.17 (1.39)	163	3.63 (1.38)	571	3.6 (1.29)	**0.001**
I was able to take my holiday entitlement											
Manager/coordinator	16	4.56 (1.09)	23	3.96 (0.88)	31	4.1 (1.35)	65	3.32 (1.68)	135	3.76 (1.49)	**0.006**
Registered nurse	35	4.8 (0.58)	11	2.55 (1.51)	68	4.15 (1.22)	14	3.5 (1.83)	128	4.12 (1.34)	**<0.001**
Nursing assistant	111	4.68 (0.81)	43	4.14 (0.97)	11	4.45 (0.93)	12	3.67 (1.67)	177	4.47 (0.98)	**0.000**
Other care staff	35	4.63 (1.06)	14	3.29 (1.33)	6	4.33 (1.63)	70	3.86 (1.43)	125	4.03 (1.39)	**0.006**
Total	199	4.69 (0.84)	92	3.77 (1.19)	116	4.17 (1.25)	162	3.6 (1.59)	569	4.13 (1.31)	**<0.001**

*n *= number; M = mean; SD = standard deviation. Significant values in bold (*p* ≤ 0.05).

**Table 4:** External support for the organization by profession and country.

	Sweden		Italy		Germany		UK		Total		*p* Value
	*n*	M (SD)	*n*	M (SD)	*n*	M (SD)	*n*	M (SD)	*n*	M (SD)	
My organization received clear guidelines											
Manager/coordinator	16	4.56 (0.63)	24	2.79 (1.28)	30	2.7 (1.34)	65	3.45 (1.35)	135	3.3 (1.38)	<**0.001**
Registered nurse	36	3.67 (0.99)	11	3.27 (1.49)	63	3.21 (1.15)	14	3.79 (1.12)	124	3.41 (1.15)	0.142
Nursing assistant	118	4.31 (0.90)	48	4.42 (0.96)	11	4.09 (0.70)	13	3.62 (1.19)	190	4.28 (0.94)	**0.044**
Other care staff	36	4.39 (0.64)	14	3.36 (1.08)	7	3.43 (1.62)	71	3.93 (1.25)	128	3.97 (1.15)	**0.014**
Total	209	4.24 (0.89)	98	3.72 (1.31)	111	3.17 (1.25)	164	3.71 (1.28)	582	3.8 (1.22)	**<0.001**
My organization was supported by the local community											
Manager/coordinator	16	4.25 (0.77)	24	3.29 (1.20)	29	2.97 (1.12)	63	3.75 (1.27)	132	3.55 (1.23)	**0.002**
Registered nurse	36	3.56 (1.03)	11	3.09 (1.30)	60	2.88 (1.24)	14	3.43 (1.16)	121	3.17 (1.20)	**0.047**
Nursing assistant	116	3.7 (1.19)	49	3.96 (1.26)	9	2.67 (1.22)	13	3.62 (1.33)	187	3.71 (1.24)	**0.036**
Other care staff	35	3.89 (1.05)	14	3 (0.88)	5	2.2 (0.84)	68	3.63 (1.26)	122	3.57 (1.20)	**0.005**
Total	206	3.75 (1.11)	99	3.56 (1.25)	103	2.85 (1.18)	159	3.67 (1.25)	567	3.53 (1.23)	**<0.001**
My organization was supported by clients’ families											
Manager/coordinator	13	3.92 (1.26)	24	3.25 (0.99)	31	3.16 (1.07)	65	4.06 (0.93)	133	3.69 (1.08)	**0.000**
Registered nurse	32	3.66 (0.83)	12	3.08 (1.24)	63	2.92 (1.14)	14	3.5 (0.76)	121	3.2 (1.08)	**0.009**
Nursing assistant	112	3.81 (1.12)	49	3.84 (1.14)	10	3.2 (0.92)	13	3.38 (1.12)	184	3.76 (1.12)	0.220
Other care staff	32	3.84 (0.88)	14	3 (1.11)	7	3.29 (1.25)	71	3.82 (1.19)	124	3.7 (1.13)	0.054
Total	192	3.8 (1.04)	100	3.48 (1.15)	111	3.04 (1.10)	164	3.86 (1.07)	567	3.61 (1.12)	**<0.001**

*n *= number; M = mean; SD = standard deviation. Significant values in bold (*p* ≤ 0.05).

**Table 5:** Factors associated with stress level among the professionals by country.

	Management		Registered Nurse		Nursing Assistant		Other Care Staff		Total	
	Coefficient	*p*-Value	Coefficient	*p*-Value	Coefficient	*p*-Value	Coefficient	*p*-Value	Coefficient	*p*-Value
Country (ref: Sweden)										
Italy	1.34 ± 0.85	0.113	1.89 ± 0.78	**0.015**	−0.14 ± 0.46	0.759	0.13 ± 0.76	0.868	0.32 ± 0.29	0.273
Germany	1.57 ± 0.85	0.064	2.12 ± 0.55	**<0.001**	−0.05 ± 0.81	0.947	0.15 ± 1.06	0.889	0.78 ± 0.29	**0.007**
UK	1.80 ± 0.78	**0.021**	3.22 ± 0.87	**<0.001**	0.98 ± 0.72	0.173	1.31 ± 0.66	**0.046**	1.59 ± 0.31	**<0.001**
Age	−0.03 ± 0.02	0.093	0.05 ± 0.02	**0.002**	0.00 ± 0.01	0.706	−0.03 ± −0.04	**0.014**	0.00 ± 0.01	0.643
Gender (ref: male)										
Female	0.47 ± 0.55	0.397	−0.46 ± 0.63	0.462	0.29 ± 0.57	0.615	0.52 ± 0.64	0.417	−1.27 ± 1.14	0.268
Organization (ref: Public organization)										
Private	0.25 ± 0.62	0.688	−0.75 ± 0.48	0.118	−0.67 ± 0.47	0.154	−0.15 ± 0.58	0.796	−0.32 ± 0.24	0.185
Non-profit	1.60 ± 0.90	0.076	−0.83 ± 0.55	0.127	0.17 ± 1.43	0.908	0.22 ± 1.10	0.844	0.35 ± 0.38	0.350
My management supported me adequately (1 point increase)	−0.68 ± 0.24	**0.005**	−0.34 ± 0.18	0.054	−0.37 ± 0.21	0.088	0.01 ± 0.27	0.972	−0.36 ± 0.10	<**0.001**
My voice has been listened to by my organization (1 point increase)	0.47 ± 0.25	**0.060**	0.13 ± 0.19	0.512	−0.04 ± 0.22	0.848	−0.24 ± 0.27	0.374	0.17 ± 0.10	0.106
I have been able to take the holiday leave I am entitled to this year (1 point increase)	−0.11 ± 0.12	0.351	−0.33 ± 0.17	**0.045**	−0.29 ± 0.19	0.122	−0.31 ± 0.16	0.061	0.32 ± 0.29	0.273

Significant values in bold (*p* ≤ 0.05).

**Table 6:** Comparison of factors affecting stress level across country.

	Support from within the Organization	External Support	Cumulative COVID-19-Related Deaths Per Million Population by the Beginning of the Study	Stress and Anxiety Level
Sweden	very high	high	high	low
Italy	high	medium	high	medium
Germany	medium	low	low	high
UK	high	high	very high	high

For support from within the organization, external support and stress and anxiety level: >4.00 = very high, 4.30–7.00 = high, 3.69–3.30 = medium, <3.30 = low. Cumulative COVID-19-related deaths by the beginning of the study: >600 = very high, 600–400 = high, 399–200 = medium, <200 = low.

## Data Availability

Data are available on reasonable request.
